# Short-Term Magnesium Supplementation Has Modest Detrimental Effects on Cycle Ergometer Exercise Performance and Skeletal Muscle Mitochondria and Negligible Effects on the Gut Microbiota: A Randomized Crossover Clinical Trial

**DOI:** 10.3390/nu17050915

**Published:** 2025-03-06

**Authors:** Matthew C. Bomar, Taylor R. Ewell, Reagan L. Brown, David M. Brown, Beatrice S. Kwarteng, Kieran S. S. Abbotts, Hannah M. Butterklee, Natasha N. B. Williams, Scott D. Wrigley, Maureen A. Walsh, Karyn L. Hamilton, David P. Thomson, Tiffany L. Weir, Christopher Bell

**Affiliations:** 1 Department of Health and Exercise Science, Colorado State University, Fort Collins, CO 80523-1582, USA; 2 Department of Food Science and Human Nutrition, Colorado State University, Fort Collins, CO 80523-1571, USA

**Keywords:** ergolytic, dietary supplement, maximal oxygen uptake, time trial, gastrointestinal microbiome

## Abstract

**Background/Objectives:** Although the importance of magnesium for overall health and physiological function is well established, its influence on exercise performance is less clear. The primary study objective was to determine the influence of short-term magnesium supplementation on cycle ergometer exercise performance. The hypothesis was that magnesium would elicit an ergogenic effect. **Methods:** A randomized, double-blind, placebo-controlled, two-period crossover design was used to study men and women who were regular exercisers. Fifteen participants ingested either a placebo or magnesium chloride (MgCl_2_ 300 mg) twice per day, for 9 days, separated by a 3-week washout. During days 8 and 9, participants completed a battery of cycle ergometer exercise tests, and whole blood, vastus lateralis, and stools were sampled. The primary outcomes were the maximal oxygen uptake (VO_2max_), a simulated 10 km time trial, and the sprint exercise performance. Additional outcomes included skeletal muscle mitochondrial respiration, and, on account of the known laxative effects of magnesium, the gut microbiota diversity. **Results:** Compared with a placebo, MgCl_2_ supplementation increased the circulating ionized Mg concentration (*p* < 0.03), decreased the VO_2max_ (44.4 ± 7.7 vs. 41.3 ± 8.0 mL/kg/min; *p* = 0.005), and decreased the mean power output during a 30 s sprint (439 ± 88 vs. 415 ± 88 W; *p* = 0.03). The 10 km time trial was unaffected (1282 ± 126 vs. 1281 ± 97 s; *p* = 0.89). In skeletal muscle, MgCl_2_ decreased mitochondrial respiration in the presence of fatty acids at complex II (*p* = 0.04). There were no significant impacts on the gut microbiota richness (CHAO1; *p* = 0.68), Shannon’s Diversity (*p* = 0.23), or the beta-diversity (Bray–Curtis distances; *p* = 0.74). **Conclusions:** In summary, magnesium supplementation had modest ergolytic effects on cycle ergometer exercise performance and mitochondrial respiration. We recommend that regular exercisers, free from hypomagnesemia, should not supplement their diet with magnesium.

## 1. Introduction

The importance of magnesium (Mg) for overall human health and physiological function is well established [1,2,3,4]. For example, magnesium plays a key role in more than 600 enzymatic reactions, including all adenosine triphosphatase (ATPase) reactions [3,5,6]. In addition, it facilitates cell biosynthesis [7], regulates glucose [4,8] and protein [9] metabolism, is integral to mitochondrial function [10], contributes to cardiovascular control [11,12], promotes bone health [13,14], and assists with the maintenance of nerve [15] and skeletal muscle [16] function.

In light of these important contributions to physiological function, it has been speculated that magnesium may be especially relevant to athletes and may possess ergogenic properties [17,18,19]. However, relative to studies of established ergogenic aids, such as caffeine and sodium bicarbonate, studies exploring the ergogenic efficacy of magnesium have generated conflicting data and underwhelming support [17,18,19]. Several possible explanations may account for these contradictory and unremarkable observations. First, it is plausible that despite its health benefits, magnesium is not ergogenic; this may be especially true in people who are not magnesium deficient. Second, there is considerable variability in the formulations of dietary magnesium supplements that have been used in exercise studies. To illustrate this, in previous studies, magnesium has been delivered as magnesium oxide [20,21,22], magnesium-L-aspartate hydrochloride [23], magnesium citrate [24,25], and as a combination of magnesium oxide and magnesium stearate [26]. Given the complexities associated with the mechanisms of absorption from the gut [1], the resulting bioavailability of magnesium in these studies is likely to differ and therefore may contribute to conflicting conclusions. Third, the quantification of the circulating concentration of the active ingredient in dietary supplements is an important aspect of ergogenic studies. The interpretation of data becomes problematic in studies of ergogenic aids if the bioavailability of the agent in question is not increased above the baseline. In this regard, controversy exists as to the best method to determine the bioavailability of magnesium [27]. Finally, and perhaps counterintuitively, increasing the dietary intake of magnesium can lead to decreased absorption and lower circulating concentrations. On account of its hypoosmotic properties, magnesium is a key component of medications commonly used to empty the intestine to prepare patients for explorative procedures, such as a colonoscopy [28]. Thus, large doses of magnesium might accelerate bowel transit, thereby decreasing the opportunity for overall nutrient absorption. Further, magnesium may influence the health of the gut via changes in the gut microbiota in a manner independent of changes in bowel transit [29].

In the current study, we attempted to avoid some of the potential limitations associated with past work. We evaluated a previously described [27,30], commercially available preparation of magnesium, magnesium chloride (MgCl_2_; ReMag^®^, New Capstone, Inc., Mooresville, NC, USA). Magnesium chloride has been shown to have greater bioavailability than magnesium oxide [31]. In addition, we quantified the magnesium bioavailability by measuring the circulating ionized magnesium; this technique has recently been suggested to be a superior and preferred method of quantification compared with the measurement of the total magnesium [27]. To maximize the external validity of our data, we studied men and women who had not been previously diagnosed with hypomagnesemia. Finally, we monitored bowel habits during the intervention to provide insight as to the potential unfavorable side effects of magnesium supplementation (e.g., diarrhea) and analyzed the gut microbiota.

The specific objectives of this study were to determine the influence of short-term magnesium supplementation, administered as magnesium chloride (MgCl_2_; ReMag^®^), on cycle ergometer exercise performance and on some of the physiological processes that may mediate exercise performance, including skeletal muscle mitochondrial respiration and the gut microbiota. The hypothesis was that magnesium would elicit an ergogenic effect.

## 2. Materials and Methods

### 2.1. Trial Design

**Figure 1:**
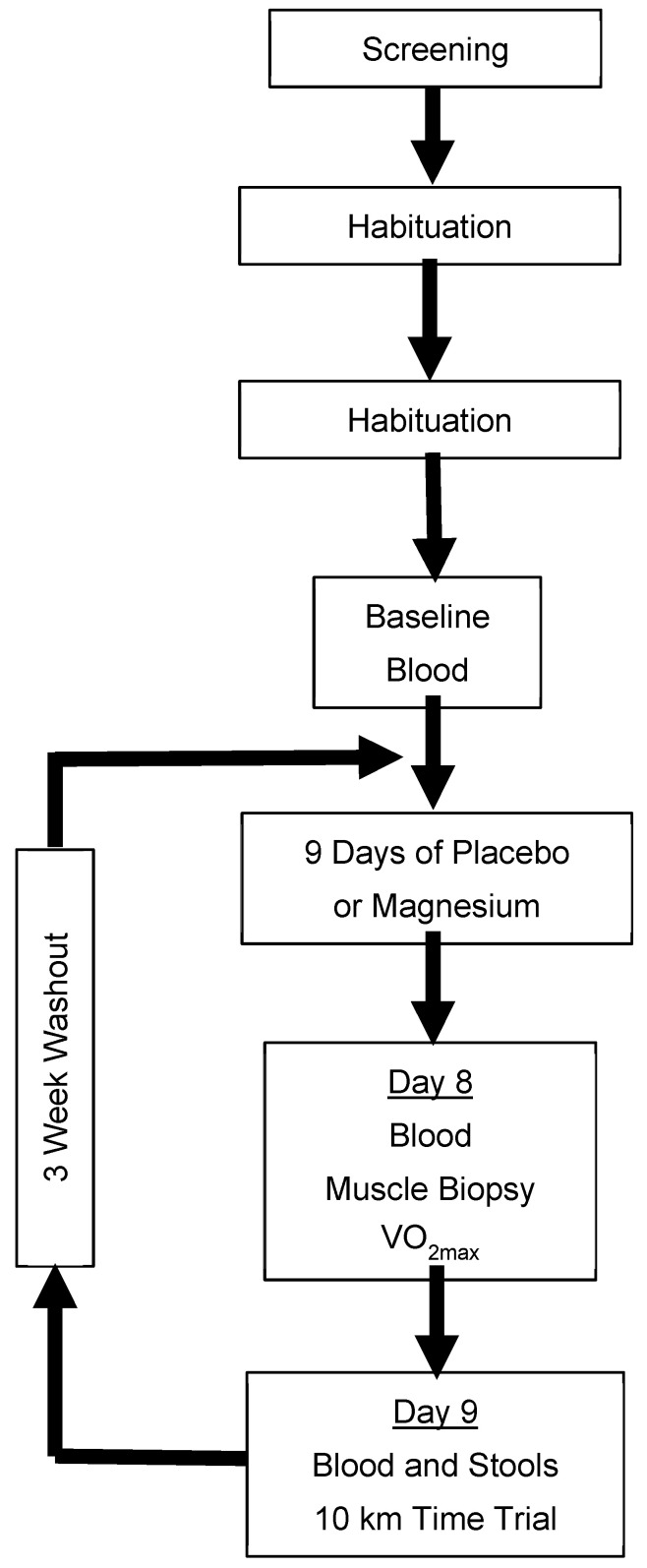
Schematical overview of the experimental design. Blood was analyzed for the ionized magnesium concentration. Placebo/magnesium was ingested every 12 h. Only participants who consented to the procedure provided samples of their vastus lateralis. VO_2max_: maximal oxygen uptake.

A randomized, double-blind, placebo-controlled, two-period crossover design was used to study males and females who were regular exercisers. Participants ingested either a placebo or magnesium chloride (MgCl_2_ 300mg; ReMag^®^) twice per day (approximately once every 12 h) for 9 days, separated by a minimum 3-week washout. During days 8 and 9, participants completed a battery of cycle ergometer exercise tests, and whole blood, skeletal muscles, and stools were sampled. The primary outcomes were the maximal oxygen uptake (VO_2max_), a simulated 10 km time trial, and the sprint exercise performance. Additional outcomes included mitochondrial respiration and the gut microbiota diversity. An overview of the trial design is presented in Figure 1.

The rationale for the use of a crossover design was to minimize the influence of between-participant variation typically associated with parallel designs [32]. The assignment order of the placebo and magnesium was randomized by M.C.B., using an online randomizer (Research Randomizer Version 4.0; https://www.randomizer.org) in a 1:1 allocation, stratified by sex. The rationale for the duration of the washout was based on a combination of the number of days required for the circulating magnesium to return to normal [33] and sufficient time for the participants to recover from the interruption of their lifestyle and the burden associated with the visits to the laboratory for physically challenging data collection (maximal exercise, etc.).

All data were collected in the laboratories of the Departments of Health and Exercise Science and Food Science and Human Nutrition at Colorado State University, Fort Collins, CO, USA.

### 2.2. Participants

This project was approved by the Institutional Review Board of Colorado State University (Protocol #21-10671H), conducted according to the guidelines of the Declaration of Helsinki, and registered as a clinical trial (NCT05282693). Written informed consent was provided by all participants prior to the initiation of any research activities. Healthy young adults were prospectively recruited by M.C.B., T.R.E., D.M.B., R.L.B., B.S.K., K.S.S.A., and H.M.B. The inclusion criteria consisted of an age between 18 and 40 years and a history of competitive cycling and/or a history of regular exercise, defined as a minimum of 5 days per week, for a minimum of 30 min per session, during the previous 2 years. The exclusion criteria included pregnancy, breastfeeding, the identification of a contraindication to exercise during an exercise stress test, the use of a magnesium supplement within the previous 4 weeks, being unable or unwilling to perform vigorous exercise, a previous diagnosis of kidney disease, and the current use of laxatives, zinc supplements, diuretics, and/or medications for treating heartburn or gut disorders that contained magnesium, zinc, or other high-dose cations known to attenuate the absorption of magnesium. During recruitment, one change was made to the inclusion criteria: an original criterion of a VO_2max_ that met the minimum classification of “Good”, as defined by the American College of Sports Medicine, was disregarded. The rationale was that many potential participants were unable to attain this classification during screening despite reporting habitual physical activity greater than 150 min per week. The moderate altitude of Fort Collins (~1525 m) may have contributed to these lower-than-expected values for the VO_2max_.

### 2.3. Screening

Potential research participants reported to the laboratory for an initial screening visit that consisted of a medical history/screening questionnaire, a venous blood sample, an assessment of their body mass, height, and body composition, and a measurement of their VO_2max_. Blood was analyzed for the circulating concentrations of markers of kidney function to identify evidence of kidney disorders. An automated device was used (Piccolo, Abaxis, Inc., Union City, CA, USA), as previously described [34]. Height was measured using a stadiometer and body mass using a physician’s scale (the body mass index was calculated from these data). The body composition was assessed using dual-energy X-ray absorptiometry (Hologic, Discovery W, QDR Series, Bedford, MA, USA) [35,36,37]. The VO_2max_ was assessed during incremental cycle ergometer exercise (20–35 W/min) to voluntary fatigue using an electro-magnetically braked ergometer (Corival CPET, Lode BV, Groningen, The Netherlands) and indirect calorimetry (ParvoMedics TrueOne 2400; Salt Lake City, UT, USA), as previously described [35,36,37,38]. During the VO_2max_ test, the beat-by-beat heart rate was monitored via 12-lead electrocardiography, and the electrocardiograms were inspected by a physician (D.P.T.) for the identification of potential contraindications to exercise.

### 2.4. Habituation

In order to familiarize the research participants with the laboratory environment, the equipment, and the protocols used for data collection, two habituation sessions were completed. These sessions were very similar to the laboratory visits described below; blood collection and placebo/magnesium ingestion were not included in the habituation sessions.

### 2.5. Interventions

Participants ingested either a placebo or magnesium chloride (300 mg MgCl_2_; ReMag^®^) twice per day (approximately once every 12 h) for 9 days, separated by a minimum 21-day washout. The interventions were delivered as lemon-flavored beverages. The placebo was identical to the magnesium supplement in appearance and taste; the only difference was the absence of magnesium chloride. Both the placebo and magnesium supplement were provided by the study sponsor (New Capstone, Inc., Mooresville, NC, USA). Certificates of analysis were provided by an independent laboratory. The bottles were stored at ~4 degrees Celsius and labeled “A” and “B”. The key required to break the codes was provided in a sealed envelope that was kept by a colleague external to the protocol. All investigators remained blind as to the identity of the interventions until the final statistical analyses were complete.

Originally, the placebo and magnesium beverages were provided in plastic bottles. Part way through the study, mold became visible inside some of the bottles. The original beverages were recalled and replaced with new beverages (same formula, etc.) but packaged in glass bottles. To the best of our knowledge, no participants ingested beverages that contained mold. No adverse events were reported.

### 2.6. Circulating Ionized Magnesium Concentration

On days 8 and 9 of the interventions, approximately 10 mL of venous blood was sampled and transferred to a serum separator tube. The tube remained at room temperature for 15 min before being transferred to a centrifuge. The serum was separated from the whole blood and placed in frozen storage (−80 °C). Following established protocols [27,39], the ionized magnesium concentration was determined in duplicate using an automated analyzer (Nova Stat Profile Prime™ ES Comp Plus Analyzer, Nova Biomedical, Waltham, MA, USA).

### 2.7. Skeletal Muscle Sampling and Mitochondrial Respiration

A subset of study participants provided additional consent to undergo the sampling of the vastus lateralis. This procedure was performed by a physician (D.P.T.) on day 8 of the intervention, following previously described protocols [36,40]. Samples were weighed and approximately ~50 mg was placed in ice-cold BIOPS (10 mM Ca-EGTA buffer, 0.1 μM free calcium, 20 mM imidazole, 20 mM taurine, 50 mM K-MES, 0.5 mM DTT, 6.56 mM MgCl_2_, 5.77 mM ATP, 15 mM phosphocreatine; pH 7.10 at 0 °C). The remaining sample was flash frozen in liquid nitrogen. Muscle fibers were prepared for high-resolution respirometry. An experienced researcher (M.A.W.) permeabilized the muscle fibers on ice using forceps. Following permeabilization, the fibers underwent chemical permeabilization in BIOPS with 12.5 µM blebbistatin and 50 µg/mL saponin for 30 min with shaking on ice, followed by a 15 min rinse with shaking on ice in BIOPS and 12.5 µM blebbistatin. Approximately 3.0 mg (wet weight) of muscle fibers were placed in a mitochondrial respiration medium (MiR05; 0.5 mM EGTA, 3 mM MgCl_2_·6H_2_O, 60 mM lactobionic acid, 20 mM taurine, 10 mM KH_2_PO_4_, 20 mM HEPES, 110 mM D-sucrose, 1 g/L fatty acid-free BSA; pH 7.10 at 21 °C) in an Oxygraph-2k (O2k) (Oroboros, Innsbruck, Austria) for high-resolution respirometry. To control for oxygen flux at higher concentrations of oxygen, each morning of the respirometry analysis, we conducted high-oxygen-concentration calibrations at 450, 350, 250, and 167 (i.e., concentration of room air) nmol/mL O_2_ [41]. During the experiments, oxygen concentrations were maintained between 225 and 450 nmol/mL O_2_. High-resolution respirometry measurements were performed in duplicate using two different protocols. A detailed explanation of the protocols is provided in Supplementary Tables S1 and S2. Mitochondrial respiration best practices were followed [42] to promote transparency and reproducibility.

Tissue was placed in each O2k chamber with subsequent hyper-oxygenation to ~450 nmol/mL O_2_. The first Substrate Uncoupler Inhibitor Titration protocol (SUIT 1) was an ADP titration protocol to test if magnesium supplementation would improve the ADP kinetics. The ADP titration determines the maximal oxidative capacity (V_max_) and ADP sensitivity (K_m_), the amount of ADP needed to achieve ½ the V_max_, and a measure of the ADP sensitivity under complex I-supported respiration. We measured complex I-supported leak respiration (State 2_(PGM)_) with the addition of 10 mM glutamate, 0.5 mM malate, and 5 mM pyruvate. Upon the acquisition of State 2_(PGM)_, we titrated progressively greater concentrations of ADP from 0.1 mM, 0.175 mM, 0.25 mM, 0.5 mM, 1 mM, 2 mM, 4 mM, 8 mM, 12 mM, and 16 mM (State 3_(PGM)_), awaiting steady-state oxygen flux prior to adding the subsequent titration to determine the complex I-linked ADP V_max_ and apparent K_m_ (i.e., ADP sensitivity). After the ADP titration was completed, we added 5 mM cytochrome C to test the mitochondrial membrane integrity. After cytochrome C addition, we added 10 mM succinate to acquire the maximal complex-I-and-II-supported coupled respiration (State 3_(PGMS)_). We then added 0.5 µM FCCP sequentially until there was no increase in respiration to determine the capacity of the electron transport system to consume oxygen, or the maximal non-coupled respiration (ETS_(CI-CIV)_). Finally, we added 5 µM rotenone to measure the maximal non-coupled respiration with the inhibition of complex I (ETS_(CII-CIV)_), followed by 2.5 µM antimycin A to measure the residual oxygen consumption (ROX) to which all other data were normalized.

The second protocol (SUIT 2) measured carbohydrate-and-fatty-acid-supported oxygen consumption. We then measured fatty acid-supported leak respiration by adding 10 mM glutamate, 0.5 mM malate, 5 mM pyruvate, and 0.2 mM octanoylcarnitine (State 2_(PGM+Oct)_) and 10 mM succinate (State 2_(PGM+Oct+S)_). After stimulating the maximal leak respiration, we added submaximal boluses of ADP (0.5 mM: State 3_(Sub+0.5D)_; 1 mM: State 3_(Sub+1.0D)_), followed by a saturating bolus of ADP (6.0 mM: State 3_(Sub+6.0D)_). We added 5 mM cytochrome C to test the mitochondrial membrane integrity. We set a cytochrome C control factor threshold of 0.15, as previously reported in human studies [43]. We added 5 µM rotenone to determine the maximal coupled respiration in the absence of complex I (State 3_[Sub+D−CI]_), followed by sequential titrations of 0.5 µM FCCP until respiration no longer increased to determine the maximal fatty acid-supported uncoupled respiration (ETS_(Sub+D−CI)_), and added 2.5 µM antimycin A to measure the ROX to which all other data were normalized.

### 2.8. VO_2max_ and Ventilatory Threshold

On day 8 of the interventions, the VO_2max_ and ventilatory thresholds were determined using protocols similar to those previously described [38]. The VO_2max_ was quantified during incremental cycle ergometer exercise (continuous ramp functions of 20–35 W/min) to voluntary fatigue using an electro-magnetically braked ergometer and indirect calorimetry. The respired gases were analyzed using an indirect calorimeter (ParvoMedics TrueOne 2400; Salt Lake City, UT, USA). The VO_2max_ was recorded as the greatest value for the VO_2_ averaged over 30 s. The ventilatory threshold was determined using established procedures [44]. Every 60 s, arterialized venous blood (~2–3 mL) was sampled from a venous catheter placed in a dorsal hand vein; the hand and forearm were wrapped in a heated blanket [45]. Blood was immediately transferred to pre-chilled tubes coated with potassium oxalate and sodium fluoride that were then returned to an ice slurry. Within 30 min of blood collection, the lactate concentration was determined using an automated analyzer (YSI 2900, Xylem Inc; White Plains, NY, USA). Gas exchange variables and the blood lactate, heart rate (via short-range telemetry; Polar T31, Bethpage, NY, USA), and ratings of perceived exertion (RPE; 6-20 Borg scale) [46] were determined throughout the test.

### 2.9. Stool Sampling and Assessment of Gut Microbiota

On day 9 of the interventions, participants provided a fecal sample. Participants also answered questions related to their perceived gut health [47]. The FastDNA SPIN Kit for stool samples (MP Biomedicals, Santa Ana, CA, USA; #116540400) was used to extract microbial DNA. Fecal DNA extracts were used to generate amplicon libraries of the 16s rRNA hypervariable V4 region (primers: 515F–806R) following Earth Microbiome Project protocols using the 515F–806R primer set containing a unique 12 bp error-correcting barcode included on the forward primer, as previously described [48,49]. Blank DNA extraction controls, negative PCR controls, and the Zymo mock gut microbial were included on the sequencing plate as quality controls. Amplicon sequencing was performed by generating 2× 250bp chemistry on an Illumina MiSeq (Illumina Inc., San Diego, CA, USA) at the Next Generation Sequencing Core Facility at Colorado State University (Fort Collins, CO, USA). The resulting 16S rRNA amplicon dataset was processed using QIIME2 (v2023.5). The cycling and sequencing conditions were as previously described [50]. Using the QIIME2 (version 2024.2) pipeline, the sequence reads were demultiplexed and concatenated. Following a quality check of the demultiplexed data using a quality score of 30 as the cutoff, the reverse reads did not meet the quality-filtering parameter. Analysis proceeded with single-end sequences and reads were truncated at 196 bp, followed by denoising and the clustering of amplicon sequence variants (ASVs) using a Dada2 single-end pipeline. Clustered ASVs were taxonomically classified using the SILVA database v138. The trimmed and denoised data were exported from QIIME2 and imported into R Studio (version 2023.03.0 +386) for further processing and analysis in MicrobiomeAnalystR (version 2.0). To remove features that may have been a result of a sequencing error or low-level contamination, a low-count filter removed reads with less than four counts and reads that were present in less than 10% of the samples; the standard deviation was used as a low-variance filter. The data were then normalized via total sum scaling. The resulting filtered and normalized data were used in downstream analyses.

### 2.10. Time Trial

On day 9 of the interventions, participants completed a time trial. Following a 5 min warm-up at a self-selected work rate, participants completed stationary cycle ergometer exercise equivalent to a distance of 10 km (6.2 miles). An air-braked stationary cycle ergometer was used (Concept2 BikeErg, Concept2 Inc., Morristown, VO, USA). Calibration was undertaken as per the manufacturer’s guidelines. Performance data were recorded electronically using software provided by the ergometer manufacturer (ErgData v 2.2.11, Concept2 Inc., Morristown, VO, USA). Participants completed this exercise as quickly as they were able. During the test, standardized verbal encouragement was provided by the research staff. The heart rate (via short-range telemetry; Polar T31, Bethpage, NY, USA) and RPE (Borg scale) [46] were determined throughout the test. To facilitate maximal performance and avoid the burden of wearing a mouthpiece and nose clips, the respired gases were not collected during the time trial.

### 2.11. Sprint Performance

On day 9 of the interventions, within 5 min of completing the time trial, participants completed a modified version of a Wingate anaerobic power test [51]. Participants were given 30 s to gradually increase their pedal revolutions to their maximal cadence. During this period, the air resistance was at the lowest setting (i.e., damper setting 1). At 30 s, the air resistance was set to the maximum (i.e., damper setting 10), and participants attempted to maintain their maximal cadence for 30 s. This “all-out” effort was considered as an indicator of their capacity for high-intensity exercise. The performance data were recorded electronically using software provided by the ergometer manufacturer (ErgData, Concept2 Inc., Morristown, VO, USA).

### 2.12. Statistical Analysis

On account of the randomized, crossover design, repeated measures statistics were utilized to compare the influence of magnesium against the placebo control. Discrete outcomes such as the VO_2max_, the VO_2_ at the ventilatory threshold, the respiratory exchange ratio, the RPE, the heart rate at the point of fatigue, the time to cycle 10 km, and the peak and mean power outputs during sprinting were compared using 2-tailed, paired Student’s *t*-tests, following the confirmation of normality via a Shapiro–Wilk test. Time-dependent variables, such as the blood lactate concentration throughout graded exercise, were compared using a 2-way (exercise time x intervention) analysis of variance with repeated measures (exercise time). The main effects and interactions were further explored using Tukey tests. Statistical tests were performed using commercially available software (SigmaStat 3.0, Systat Software Inc., San Jose, CA, USA). Significance was defined a priori as *p* < 0.05. All data are expressed as the mean and standard deviation.

For mitochondrial respirometry, in line with best practices, technical replicates were averaged. The apparent K_m_ and V_max_ values were determined using Michaelis–Menten kinetics for each individual O2k run in Prism 9.0 (La Jolla, CA, USA) as previously described [52,53]. The data are presented as the mean ± the standard deviation (SD), and significance was set a priori at *p* < 0.05. A subset of the study participants consented to the muscle sampling procedure. Accordingly, this relatively smaller study population was underpowered to detect differences in mitochondrial oxygen consumption. For this reason, statistical trends (*p* < 0.10) are also reported. A ROUT outlier test was used to identify and remove any statistical outliers. There were no outliers. Normality was confirmed by the Shapiro–Wilk test. Student’s *t*-tests were used to determine the treatment differences.

For the microbiome analysis, the alpha diversity measures (Shannon index and Chao1) for each treatment were calculated in R, and statistically significant differences were determined using Mann–Whitney post hoc tests with Benjamini–Hochberg adjustment for false discovery (FDR). A Principal Coordinate Analysis (PCoA) based on Bray–Curtis dissimilarity metrics was used to observe differences in bacterial communities between the magnesium and placebo arms of the study. Differences in the Bray–Curtis distances were statistically analyzed using a permutational analysis of variance (PERMANOVA) as well as pairwise PERMANOVAs. The differential abundance of specific taxa between the magnesium and placebo treatments was determined using Microbiome Multivariable Association with Linear Models (MaAsLin2), incorporating the subject as a covariate, and fitted to the linear model with adjusted *p*-value set to 0.05.

## 3. Results

**Figure 2:**
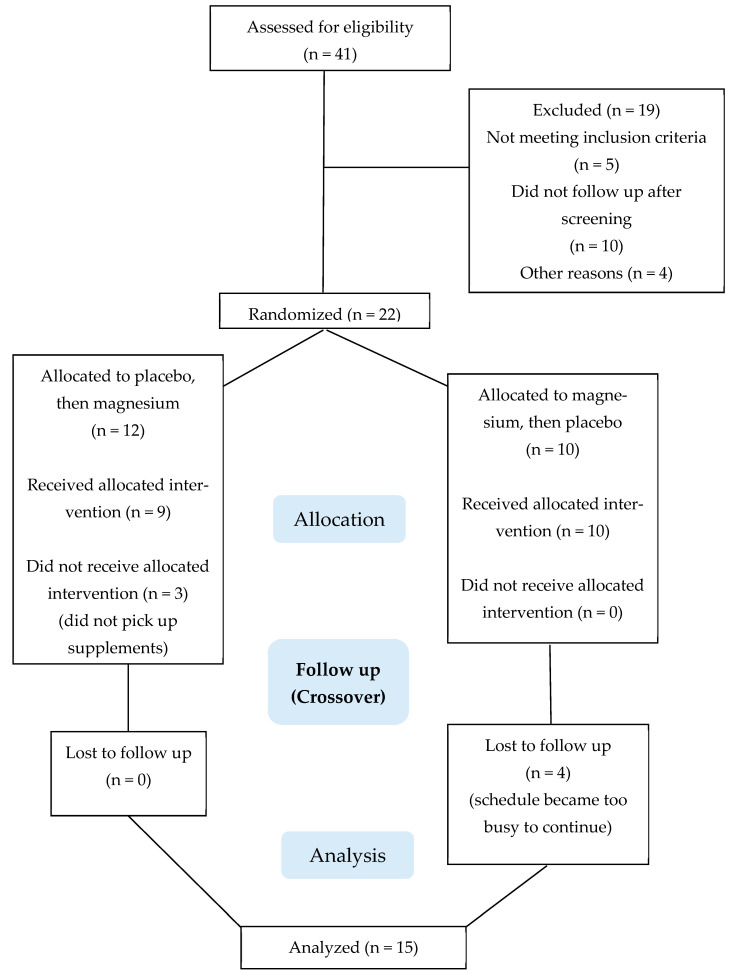
Consolidated Standards of Reporting Trials (CONSORT) flow diagram.

The recruitment of participants and collection and analysis of data took place between December 2021 and November 2024. Participant recruitment and testing was paused between June 2022 and November 2022 while the original magnesium and placebo mixtures were recalled and replaced with fresh preparations. Figure 2 summarizes the participant flow through the protocol. A total of 41 people were assessed for study eligibility, of which 22 were enrolled as participants. Fifteen participants completed the project; seven ended their participation early, primarily for reasons pertaining to scheduling conflicts and the perceived time commitment required for study participation. There were no adverse events.

Of the 15 study participants who completed the study, 8 were male and 7 were female. The baseline characteristics of the body composition appeared consistent for healthy, young, regularly exercising adults: an age of 26 ± 5 years; body mass of 70.7 ± 11.4 kg; body mass index of 24.6 ± 3.2 kg/m^2^; fat mass of 18.5 ± 5.5 kg; lean mass of 49.9 ± 8.9 kg; and body fat of 25.9 ± 5.6%. The markers of kidney function were normal (13 ± 5 mg/dL blood urea nitrogen, 1.0 ± 0.3 mg/dL creatinine, 0.9 ± 0.3 mg/dL total bilirubin, 4.0 ± 0.2 g/dL albumin, and 7.5 ± 0.5 g/dL total protein).

### 3.1. Circulating Ionized Magnesium Concentration

Compared with the baseline (0.47 ± 0.20 mmol/L), the circulating ionized magnesium concentration was increased after 8 (0.63 ± 0.09 mmol/L; *p* = 0.015) and 9 (0.61 ± 0.08 mmol/L; *p* = 0.033) days of magnesium supplementation. In contrast, the circulating ionized magnesium concentration was not different from the baseline following the ingestion of the placebo (0.53 ± 0.16 and 0.59 ± 0.03 mmol/L, days 8 and 9, respectively).

### 3.2. Responses to Incremental Cycle Ergometer Exercise

**Figure 3:**
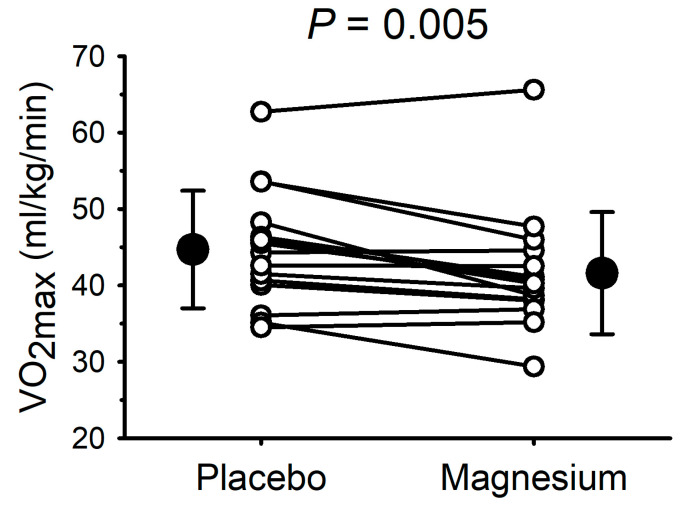
Short-term magnesium chloride supplementation decreased maximal oxygen uptake. White circles and connecting lines represent individual data (n = 15). Solid circles and errors bars represent mean and standard deviation.

The VO_2max_ data are presented in Figure 3. Compared with the placebo, the VO_2max_ was lower (*p* = 0.005) after magnesium supplementation in 11/15 participants (mean decrease: 3.1 ± 3.6 mL/kg/min). Consistent with the data illustrated in Figure 3, the absolute VO_2max_ (i.e., not normalized to the body mass) was also lower with magnesium (3.21 ± 0.76 with the placebo vs. 2.99 ± 0.72 L/min with magnesium; *p* = 0.01). At the point of fatigue, there were no differences in the respiratory exchange ratio (1.35 ± 0.21 with the placebo vs. 1.26 ± 0.17 with magnesium; *p* = 0.14), RPE (18 ± 2 with the placebo vs. 18 ± 2 with magnesium; *p* = 1.0), or heart rate (181 ± 9 with the placebo vs. 179 ± 10 beats/min with magnesium; *p* = 0.46). The VO_2_ at the ventilatory threshold was also unaffected by magnesium, whether it was expressed as the absolute VO_2_ (1.53 ± 0.27 with the placebo vs. 1.52 ± 0.31 L/min with magnesium; *p* = 0.88) or as a percentage of the VO_2max_ (49 ± 8 with the placebo vs. 51 ± 4% with magnesium; *p* = 0.13). The blood lactate concentration was increased with the work rate (main effect of the work rate, *p* < 0.01) but was unaffected by magnesium (interaction between the work rate and the placebo vs. magnesium, *p* > 0.05).

### 3.3. Cycle Ergometer Time Trial Performance

The time taken to complete the 10 km time trial was not different (*p* = 0.89) between the placebo (1282 ± 126 s) and magnesium (1281 ± 97 s). Throughout the time trial, the average work rate (174 ± 44 with the placebo vs. 172 ± 39 W with magnesium; *p* = 0.39), heart rate (173 ± 11 with the placebo vs. 175 ± 11 beats/min with magnesium; *p* = 0.94), and RPE (16 ± 2 with the placebo vs. 16 ± 1 with magnesium; *p* = 0.95) were also not different.

### 3.4. Cycle Ergometer Sprint Performance

**Figure 4:**
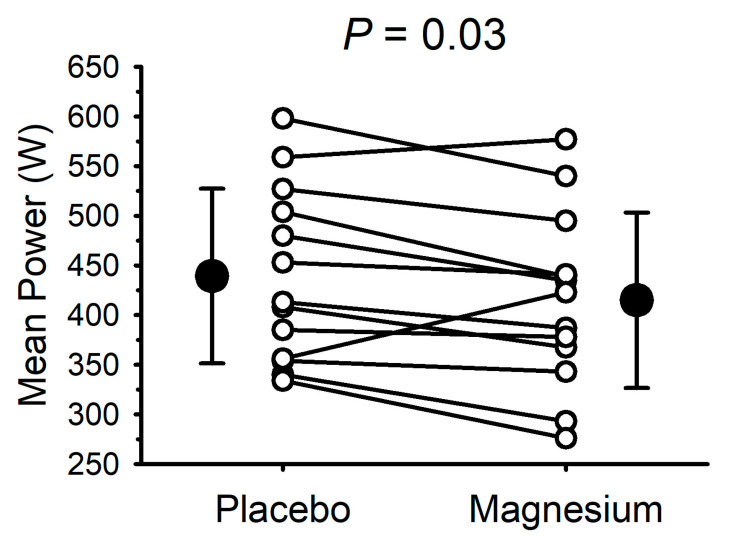
Short-term magnesium chloride supplementation decreased mean power during 30 s cycle ergometer sprint. White circles and connecting lines represent individual data (n = 13). Solid circles and errors bars represent mean and standard deviation.

Due to technical issues, complete data collection (i.e., for the placebo and magnesium) was only possible for 13 participants (7 males and 6 females). The mean power output during the 30 s cycle ergometer sprint test is presented in Figure 4. Compared with the placebo, the mean power output was lower (*p* = 0.03) after magnesium supplementation in 11/13 participants (mean decrease of 24 ± 36 W). The peak power was unaffected by magnesium (655 ± 150 with the placebo vs. 665 ± 185 W with magnesium; *p* = 0.70).

### 3.5. Gut Health and Microbiota

Based on responses to the perceived gut health questionnaire, there were two reports of mild nausea (placebo: one; magnesium: one), one report of mild heartburn (placebo: one; magnesium: zero), one report of moderate heartburn (placebo: zero; magnesium: one), four reports of mild abdominal pain (placebo: one; magnesium: three), five reports of a mild headache (placebo: one; magnesium: four), one report of a moderate headache (placebo: zero; magnesium: one), five reports of mild diarrhea (placebo: one; magnesium: four), four reports of moderate diarrhea (placebo: one; magnesium: three), one report of severe diarrhea (placebo: zero; magnesium: one), one report of mild constipation (placebo: zero; magnesium: one), and one report of moderate constipation (placebo: one; magnesium: zero).

**Figure 5:**
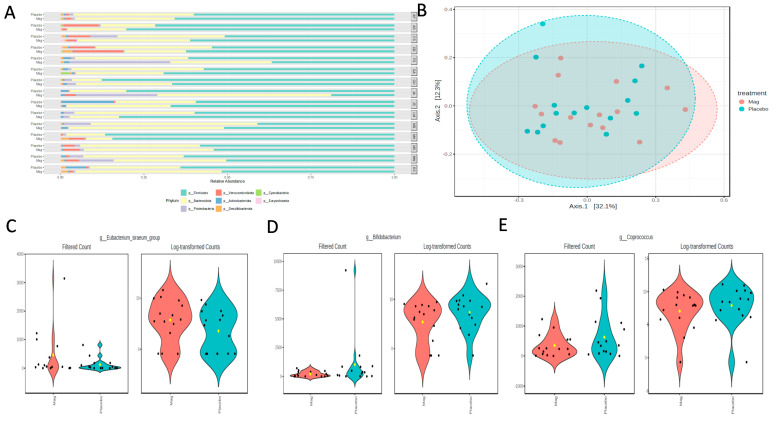
(**A**) Phyla-level relative abundances of taxa for each participant during the placebo or magnesium (Mag) intervention periods. (**B**) Bray–Curtis distances visualized in a Principal Coordinates Analysis (PCoA) showed no significant clustering by treatment group. A few taxa were differentially abundant between the treatment groups, as determined using the linear model in MaAsLin2. (**C**) *Eubacterium siraeum* increased with magnesium, and (**D**) *Bifidobacterium* and (**E**) *Coprococcus* decreased with magnesium.

The microbiota analysis was based on an average read count of 54,708 per sample (min = 31,536; max = 54,708). The major phyla detected included Bacillidota (formerly Firmicutes), Bacteriodota (formerly Bacteroidetes), Psuedomonadota (formerly Proteobacteria), Verrucomicrobiota, and Actinomycetota (formerly Actinomycetes), with Desulfobacterota, Cyanobacteria, and Euryarchaeota occurring as minor phyla in some individuals. Although there were no significant overall shifts in the phyla over the treatment period, there were some notable shifts at the individual level (Figure 5A). The most consistently observed changes were an expansion in the relative abundance of Psuedomonadota and a reduction in Actinomycetota. However, magnesium consumption had no significant effect on the alpha diversity parameters of the microbiota richness (CHAO1; *p* = 0.68) or diversity (Shannon’s index; *p* = 0.23). Consequently, there were no shifts in the Bray–Curtis distances between the treatment regimens (PERMANOVA; *p* = 0.74; Figure 5B). However, there were a few significantly different genera detected using MaAsLin2 fitted to the linear model. Specifically, the Eubacterium siraeum group was increased with magnesium (LogFC = 1.54; FDR = 0.024; Figure 5C), and both Bifidobacterium (LogFC = -1.76; FDR = 0.049; Figure 5D) and Coprococcus (LogFC = −0.93; FDR = 0.047; Figure 5E) decreased with magnesium.

### 3.6. Skeletal Muscle (Vastus Lateralis) Mitochondrial Respiration

**Figure 6:**
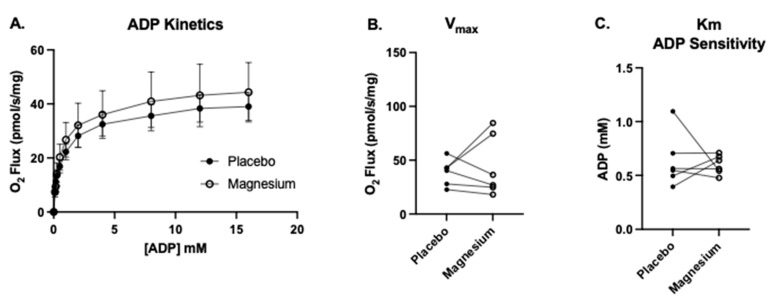
No treatment differences in the maximal oxygen flux (Vmax) or ADP sensitivity (Km). We measured complex I-supported leak respiration (State 2_(PGM)_) with the addition of glutamate, malate, and pyruvate. (**A**) Upon the acquisition of State 2_(PGM)_, we titrated progressively greater concentrations of ADP from 0.1 to 16 mM (State 3_(PGM)_) to determine (**B**) the complex I-linked ADP V_max_ and (**C**) apparent K_m_ (the amount of ADP required to achieve ½ the V_max_, a measure of the ADP sensitivity under complex I-supported respiration).

Six participants (four males and two females) consented to the muscle biopsy procedure. The first SUIT protocol was carried out to assess the treatment differences in complex I-linked mitochondrial oxygen consumption in response to carbohydrate substrates (pyruvate, glutamate, and malate), followed by the titration of a range of submaximal through saturating ADP concentrations. This protocol also enabled the assessment of the apparent maximal oxygen consumption (V_max_) and the ADP sensitivity (Km). As shown in Figure 6, there were no treatment differences in the V_max_ or K_m_.

**Figure 7:**
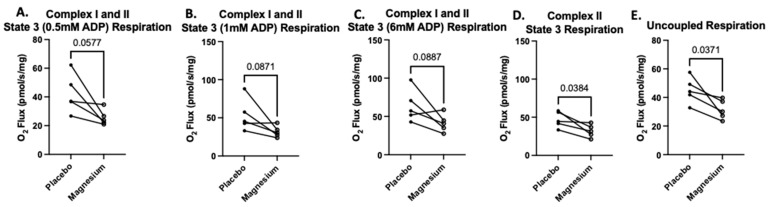
Treatment with magnesium decreased carbohydrate-and-fatty-acid-supported oxygen consumption. The second SUIT protocol was designed to assess carbohydrate-and-fatty-acid-supported oxygen consumption via titrations of glutamate, malate, pyruvate, octanoylcarnitine, and succinate (State 2 respiration). There were no treatment differences in State 2 respiration. To assess State 3 respiration, we added submaximal boluses of ADP. At all concentrations of ADP (**A**–**C**), the magnesium treatment group either tended to have lower respiration or was associated with significantly lower respiration compared to the placebo group. When we added rotenone to determine the maximal respiration in the absence of complex I (**D**), we found that respiration was significantly lower in the magnesium group compared to the placebo group (**D**). Finally, after FCCP was added to uncouple the electron transfer system from oxidative phosphorylation, respiration was significantly lower in the magnesium group compared to the placebo group (**E**).

The second SUIT protocol was designed to assess carbohydrate-and-fatty-acid-supported oxygen consumption. There were no treatment differences in State 2 respiration (oxygen consumption in response to pyruvate, glutamate, malate, octanoylcarnitine, and succinate but no ADP). However, when submaximal and saturating concentrations of ADP were added, State 3 respiration was either significantly lower or tended to be lower in the magnesium group compared to the placebo group (Figure 7A–C). When rotenone was added to assess respiration in the absence of complex I, respiration was significantly lower in the magnesium group compared to the placebo group (Figure 7D). Finally, when FCCP was added to uncouple the electron transfer system from oxidative phosphorylation, respiration was significantly lower in the magnesium group compared to the placebo group (Figure 7E).

## 4. Discussion

The major findings of the current study were that, compared with a placebo, the short-term supplementation of magnesium chloride decreased the VO_2max_, mean power during a 30 s sprint, and skeletal muscle mitochondrial respiration in the presence of fatty acids at complex II. In addition, short-term magnesium supplementation did not appreciably influence the 10 km cycle ergometer time trial performance or the gut microbiota.

The clinical criterion for hypomagnesemia is a circulating serum concentration lower than 0.7 mmol/L (1.7 mg/dL) [1]. It has been estimated that the prevalence of hypomagnesemia may be as high as 10% of the general population [1] and approximately 70% of patients admitted to intensive care units [54]. In the current study, we measured the ionized magnesium concentration, proposed by some to be the preferred marker of the magnesium status [27,55]. None of the participants had baseline ionized magnesium concentrations that would be considered outside of a normal reference range [56,57] or potentially suggestive of hypomagnesemia. In addition, the circulating concentrations of ionized magnesium following the short-term intervention were comparable with a previous human study involving the ingestion of magnesium chloride [27]. Thus, in the context of magnesium, our study participants appeared unremarkable, and the short-term dietary supplementation of magnesium increased the circulating ionized magnesium concentration in a manner consistent with previous supplement studies.

The VO_2max_ is considered an important determinant of endurance exercise performance. In addition, it is predictive of mortality [58,59,60]; thus, it has relevance to athletic populations and for public health at large. The VO_2max_ is determined by the product of the cardiac output (i.e., central oxygen delivery) and arterio-venous oxygen difference (i.e., oxygen utilization). In light of the contribution of magnesium to the regulation of the cardiovascular system [11,12] and skeletal muscle function [16] and its facilitative role in bioenergetics, it seems plausible that dietary magnesium supplementation may increase the VO_2max_. Thus, the modest decrease we observed in the current study was unexpected. Although the magnitude of the decline was modest (6.7%), based on previous studies in our lab [61], the coefficient of variation for this measurement was 3.1%; therefore, the decrease appears both statistically and physiologically significant and unlikely to be attributed to chance. Our observation of a magnesium-mediated decrease in the VO_2max_ is consistent with at least two previous lines of inquiry. In postmenopausal women, the VO_2peak_ was increased following dietary magnesium depletion and returned to the baseline (i.e., was decreased) when dietary magnesium was repleted [62]. In neurally intact dogs, the intravenous delivery of magnesium chloride decreased the cardiac output, in part by lowering the heart rate and left ventricular contractility [63]. When the heart rate was controlled and the experiment repeated, the cardiac output remained lower with magnesium. Although in the current study the maximal heart rate was not different between the placebo and magnesium, it is possible that the decrease in the VO_2max_ could be partially explained by a decreased cardiac output. We acknowledge that in humans, the heart rate may not always reflect other determinants of the cardiac output, including the stroke volume [64]. Alternatively, in patients with stable coronary artery disease, 6 months of dietary supplementation with Magnosolv-Granulat (Viatris Arzneimittel, Vienna, Austria) increased the VO_2max_ and left ventricular ejection fraction compared with a placebo [65]. Differences between this and the current study include the health of the study participants (i.e., healthy vs. coronary artery disease), the age of the study participants (26 vs. 61 years), the duration of the intervention (9 days vs. 6 months), and the formulation of the magnesium (magnesium chloride vs. magnesium citrate). Given these important differences, an explanation to account for the conflicting outcomes between the studies is likely to be multifactorial. At least three other studies have explored the influence of magnesium on the VO_2max_ in healthy, young, regularly exercising adults [66,67,68] and one in adults with chronic obstructive pulmonary disease [69]; none of these studies reported magnesium-mediated changes in the VO_2max_. Noteworthy, and consistent with the current findings, one of these previous studies also demonstrated no effect of magnesium on the VO_2_ at the ventilatory threshold [66].

Despite the decrease in the VO_2max_, magnesium had no effect on our test of endurance performance: the 10 km time trial. Several other studies have also reported negligible influences of magnesium on endurance exercise performance, including marathon running performance [23] and 20 km stationary cycle ergometer time trials [70]. The dissociation of the VO_2max_ and endurance performance in the current study may pertain to the duration of the time trial (~20 min). That is, a longer duration challenge, more reliant on the aerobic capacity, conceivably could have revealed an unfavorable modification consistent with the decrease in the VO_2max_. Nevertheless, the observations from the current study appear to be supported by previous reports that magnesium supplementation does not improve endurance exercise performance.

In contrast to a negligible effect on endurance exercise, in the current study magnesium decreased the mean power output during a sprint test. Previous studies examining the influence of magnesium on sprint and anaerobic exercise performance have yielded mixed results, with some reporting improved repeated sprint running [71] and an improved ability to perform brief and explosive activities (e.g., countermovement jump) [67], while others reported negligible effects [66]. In our 30 s cycle ergometer test, magnesium lowered the mean power output in 11/13 participants; thus, this unfavorable effect appeared reasonably consistent across individuals. Differences between the conclusions of the current and previous studies may be in part attributable to the exercise modality (i.e., cycling vs. running and/or jumping).

As has been reviewed extensively [10], magnesium is known to serve important roles in the regulation of calcium handling and a host of enzymes, including critical mitochondrial enzymes such as dehydrogenases and F0/F1-ATP synthase in oxidative phosphorylation [6]. Further, magnesium plays many regulatory roles pertaining to enzyme activities in the tricarboxylic acid cycle and oxidative phosphorylation [11,72,73], and the mitochondria serve as an intracellular storage depot for magnesium [74]. Therefore, it was logical to hypothesize that supplementation with magnesium may be of benefit for skeletal muscle mitochondrial function. To complement our assessment of the whole-body VO_2max_, we used high-resolution respirometry to measure mitochondrial oxygen consumption in permeabilized bundles of skeletal muscle fibers. This approach to assessing mitochondrial respiration is advantageous compared to making measurements in isolated mitochondria in that it facilitates evaluation in intact mitochondrial networks with the preservation of essential cellular interactions and signaling events that may influence mitochondrial respiration [42,75]. Contrary to our hypothesis, magnesium supplementation tended to or significantly decreased carbohydrate-and-fatty-acid-supported State 3 mitochondrial respiration at submaximal and saturating ADP concentrations. This difference persisted when complex I was inhibited. Further, when we chemically uncoupled the electron transfer system from oxidative phosphorylation, there was still a lower rate of mitochondrial oxygen consumption in the samples from the magnesium-supplemented condition compared to the placebo condition. These data are in contrast to a pre-clinical study reporting that magnesium supplementation improves cardiac muscle mitochondrial ATP production in a mouse model of diabetes [76]. Species and health status differences aside, to our knowledge, the current study presents the first evidence that short-term magnesium supplementation in healthy, active, young adult humans may be detrimental to skeletal muscle mitochondrial respiration. We acknowledge the limitation of our relatively small sample size and short period of supplementation and suggest that our data and initial observations could be used for power and sample size calculations for future studies.

Finally, we considered the effects of magnesium supplementation on the gut microbiota composition. Most evidence for gut microbiota modulation by magnesium supplementation comes from rodent models. In one study, rats were fed one of three diets, a control (1000 mg/kg magnesium), low-magnesium (60 mg/kg), or high-magnesium (6000 mg/kg) diet, for two weeks [77]. The control and low-magnesium diet groups had higher microbial diversity than the high-magnesium group and an increase in Proteobacteria (now called Pseudomonadota) and Parabacteroides. Although we did not observe a consistent increase in Pseudomonadota with magnesium supplementation, several individuals did show a dramatic expansion in the relative abundance of this phylum. Although an increased relative abundance of Pseudomonadota is associated with gut dysbiosis, there were no differences in the alpha or beta-diversity parameters in our study, suggesting the impacts on the gut microbiota were minimal.

We observed some changes in specific genera after 8 days of magnesium supplementation. Most notably, there was a ~1.7 log-fold decrease in Bifidobacterium, a common beneficial member of the gut milieu. Although similar changes in Bifidobacterium have not been previously reported with magnesium supplementation, mice fed a magnesium-deficient diet for 4 days showed a 1.5 log-fold decrease in Bifidobacterium, accompanied by markers indicative of reduced gut barrier function and increased inflammation [78]. Interestingly, after 21 days of magnesium deficiency, these changes reverted, which the authors suggest was due to the metabolic flexibility of these organisms, allowing them to adapt and recover by altering metabolic pathways for fermentation. Alternatively, these shifts may occur independent of changes in dietary magnesium, simply representing natural population flux in microbial community dynamics. Thus, it is difficult to interpret the significance of our observed decrease in Bifidobacteria with magnesium supplementation. While this may be associated with reductions in the gut barrier integrity, the short-term nature of this study and lack of longitudinal sample collection prevent the determination of whether this represents a transient change as the body adapts to the intervention. Likewise, the other taxa shifts that we observed after magnesium supplementation are also difficult to interpret in terms of their impacts on human health or physiology. Higher levels of Eubacterium siraeum, as we observed with magnesium intake, have been associated with reduced fat accumulation in mice [79] and improved lipid parameters in non-human primates [80]. In contrast, we also observed decreases in Coprococcus, which is a butyrate-producing microbe implicated in better health outcomes [81]. Overall, the changes that we observed were minimal, but longitudinal sampling, longer supplementation times, and perhaps a larger sample size would be needed to establish whether there are any lasting positive or negative impacts on the gut microbiota and associated health outcomes.

There are several limitations of our study that should be acknowledged, which in turn may contribute to recommendations for future studies. As previously highlighted, our study could have benefitted from a larger sample size and longer supplementation period. In addition, attention to dietary intake may have been useful, as would have consideration of the potential for a genetic predisposition to influence magnesium metabolism. While we believe some of these limitations to be beyond the scope of our current study, we recognize their potential usefulness in future research.

## 5. Conclusions

In summary, in healthy young adults who were regular exercisers and free from hypomagnesemia, short-term dietary magnesium supplementation had modest ergolytic effects, evidenced by a decreased VO_2max_, decreased mean power output during a sprint test, and decreased skeletal muscle mitochondrial respiration. The influence on the 10 km time trial performance was negligible. We recommend that regular exercisers, free from hypomagnesemia, should focus on eating high-quality, nutritious foods and refrain from supplementing their diet with magnesium.

## Data Availability

The data presented in this study are available on request from the corresponding authors. The data are not publicly available due to privacy reasons. The gut microbiota data are available at https://qiita.ucsd.edu/study/description/15910. A Qiita account is required to access these data. A free account can be created here: https://qiita.ucsd.edu/auth/create/.

## Supplementary Materials

The following supporting information can be downloaded at: https://www.mdpi.com/article/10.3390/nu17050915/s1, Supplementary Table S1: Summary of high resolution respirometry experiment using Ouroboros Oxygraph-2000. A table highlighting SUIT 1 protocol used in the study. Further explanations of these respiratory states and measurements can be found in Pesta & Gnaiger, 2011 or at the Ouroboros website: www.bioblast.at. Supplementary Table S2 Summary of high resolution respirometry experiment using Ouroboros Oxygraph-2000. A table highlighting SUIT 2 protocol used in the study. Further explanations of these respiratory states and measurements can be found in Pesta & Gnaiger, 2011 or at the Ouroboros website: www.bioblast.at. Abbreviations: P—pyruvate; G—glutamate; M—malate; S—succinate; Oct—octanoylcarnitine; ADP—adenosine diphosphate; Cyt C—cytochrome C; ATP—adenosine triphosphate; Rot—rotenone; FCCP—Trifluoromethoxy carbonylcyanide phenylhydrazone; Ama—Antimycin A; ETS—electron transport system; Sub—priorly added substrates (i.e. pyruvate, glutamate, malate, octanoylcarnitine, and succinate); CI—Complex I; CII—Complex II; CIII—Complex III; CIV—Complex IV; CV—Complex V or ATP Synthase; ROX—residual oxygen consumption; RCR—respiratory control ratio; NADH—reduced nicotinamide adenine dinucleotide.
