# Supplementary material for: Short-Term Magnesium Supplementation Has Modest Detrimental Effects on Cycle Ergometer Exercise Performance and Skeletal Muscle Mitochondria and Negligible Effects on the Gut Microbiota: A Randomized Crossover Clinical Trial
Source: Nutrients. 2025 Mar 6;17(5):915. doi: 10.3390/nu17050915 (PMC11901567; doi:10.3390/nu17050915)
Supplement: Supplementary file 1 [file nutrients-17-00915-s001.zip › nutrients-3505215-supplementary.pdf]

Supplementary Table S1 Summary of high resolution respirometry experiment using Oroboros Instruments' Oxygraph-2K. A table highlighting SUIT 1 protocol used in the study.

Supplementary Table S2 Summary of high resolution respirometry experiment using Oroboros Instruments' Oxygraph-2K. A table highlighting SUIT 2 protocol used in the study.

Abbreviations: P – pyruvate; G – glutamate; M – malate; S – succinate; Oct – octanoylcarnitine; ADP – adenosine diphosphate; Cyt C – cytochrome C; ATP – adenosine triphosphate; Rot – rotenone; FCCP – Trifluoromethoxy carbonylcyanide phenylhydrazone; Ama – Antimycin A; ETS – electron transport system; Sub – priorly added substrates (i.e. pyruvate, glutamate, malate, octanoylcarnitine, and succinate); CI – Complex I; CII – Complex II; CIII – Complex III; CIV – Complex IV; CV – Complex V or ATP Synthase; ROX – residual oxygen consumption; RCR – respiratory control ratio; NADH – reduced nicotinamide adenine dinucleotide

Supplementary Table S1

Respiratory State	Substrates	Description
**SUIT 1 – A Complex I supported ADP titration evaluating ADP sensitivity as well as maximal respiration, followed with uncoupled respiration.**		
**State 2 [PGM]**	5 mM pyruvate (P), 10 mM glutamate (G), 0.5 mM malate (M)	LEAK respiration in the presence of Complex I substrates. The amount of oxygen consumption as a consequence of protons leaking across the membrane.
**State 3 [PGM]**	Previous steps + addition of ADP such that [ADP] progressively increased as follows: 0.1 mM, 0.175 mM, 0.25 mM, 0.5 mM, 1 mM, 2 mM, 4 mM, 8 mM, 12 mM, 16 mM	Respiration associated with ATP production. NADH is generated through the oxidation of PGM, donating electrons to Complex I, and creating a proton gradient where ADP is the limiting factor for ATP production. The amount of oxygen consumed is a consequence of ADP being phosphorylated to ATP. Initial titrations are sub-saturating concentrations of ADP increasing to maximal ADP stimulated respiration.
**State 3 [Cyt C]**	Previous step + 5 mM Cytochrome C	During permeabilization, the outer membrane of mitochondria can be damaged, resulting in loss of cytochrome C. An increase in oxygen consumption is indicative of cytochrome C release during mechanical or chemical permeabilization.
**State 3 [PGM + S]**	Previous step + 10 mM succinate (S)	Same as previous step but now both NADH and $FADH_2$ are generated through the oxidation of Complexes I (PGM) and II (S) substrates yielding maximal ADP stimulated respiration.
**ETS [CI – CIV]**	Previous step + 1.0 mM FCCP	FCCP is a protonophore allowing hydrogen ions through the inner-mitochondrial membrane, uncoupling the proton gradient from ATP production through ATP synthase. This reflects the maximal or reserve capacity of Complexes I – IV, unconstrained from ATP synthase activity.
**ETS [CII – CIV]**	Previous step + 5 µM rotenone (Rot)	Rotenone is an inhibitor of Complex I. Therefore, uncoupled oxygen consumption is indicative of Complex II – IV capacity.
**ROX**	2.5 µM Antimycin A (Ama)	Antimycin A inhibits Complex III thus shutting down the electron transfer pathway. Residual oxygen consumption (ROX) is the remaining oxygen consumed from other pathways independent of Complex IV.

Supplementary Table S2

**SUIT 2 – A protocol measuring coupled and uncoupled respiration in the presence of carbohydrates and fatty acids.**		
**State 2 [PGM + Oct]**	5 mM pyruvate (P), 10 mM glutamate (G), 0.5, mM malate (M), 0.2 mM octanoylcarnitine (Oct)	LEAK respiration in the presence of Complex I and II substrates including both carbohydrates and fatty acids. The amount of oxygen consumption as a consequence of protons leaking across the membrane.
**State 2 [PGM + Oct + S]**	Previous step + 10 mM succinate (S)	Same as previous step with the addition of the Complex II substrate, succinate (S). This is considered to stimulate maximal LEAK respiration as well as generation of reactive oxygen species (ROS).
**State 3 [Sub + 0.5D]**	Previous step + 0.5 mM ADP	Respiration associated with ATP production. NADH and $FADH_2$ is generated through the oxidation of PGMS and octanoylcarnitine donating electrons to Complexes I and II, and creating a proton gradient where ADP is the limiting factor for ATP production. The amount of oxygen consumed is a consequence of ADP being phosphorylated to ATP with a sub-saturating bolus of ADP.
**State 3 [Sub + 1.0D]**	Previous step + 0.5 mM ADP	Same as previous step. The final concentration of ADP is higher but still sub-saturating stimulated respiration.
**State 3 [Sub + 6.0D]**	Previous step + 5 mM ADP	Same as previous step, however, the final concentration of ADP (6.0 mM) is a saturating does, maximally stimulating mitochondrial respiration and ATP production.
**State 3 [Cyt C]**	Previous step + 5 mM Cytochrome C	During permeabilization, the outer membrane of mitochondria can be damaged, resulting in loss of cytochrome C. An increase in oxygen consumption is indicative of cytochrome C release during mechanical or chemical permeabilization. After the addition of cytochrome C, no more assessments of ROS emission were measured.
**State 3 [Sub + D – CI]**	Previous step + 5 µM rotenone (Rot)	Rotenone is an inhibitor of Complex I. Oxygen consumption is due to maximally stimulated ADP respiration of Complexes II – IV.
**ETS [Sub + D – CI]**	Previous step + 1.0 mM FCCP	FCCP is a protonophore allowing hydrogen ions through the inner-mitochondrial membrane, uncoupling the proton gradient from ATP production through ATP synthase. This is the maximal or reserve capacity of the mitochondria for Complexes II – IV.
